# Analysis of Prognostic Factors for Resected Synchronous and Metachronous Liver Metastases from Colorectal Cancer

**DOI:** 10.1155/2018/5353727

**Published:** 2018-07-11

**Authors:** Ilenia Bartolini, Maria Novella Ringressi, Filippo Melli, Matteo Risaliti, Marco Brugia, Enrico Mini, Giacomo Batignani, Paolo Bechi, Luca Boni, Antonio Taddei

**Affiliations:** ^1^Department of Surgery and Translational Medicine, University of Florence, AOU Careggi, Largo Brambilla 3, 50134 Florence, Italy; ^2^Department of Experimental and Clinical Medicine, AOU Careggi, Largo Brambilla 3, 50134 Florence, Italy; ^3^Clinical Trials Coordinating Center of Istituto Toscano Tumori, AOU Careggi, Largo Brambilla 3, 50134 Florence, Italy

## Abstract

**Background:**

Surgical treatment is the cornerstone in the management of colorectal cancer (CRC) liver metastases. The aim of this study is to identify clinicopathological factors affecting disease-free (DFS) and overall survival (OS) in patients undergoing potentially curative liver resection for CRC metastasis.

**Methods:**

All consecutive patients undergoing liver resection for first recurrence of CRC from February 2006 to February 2018 were included. Prognostic impact of factors related to the patient, primary and metastatic tumors, was retrospectively tested through univariate and multivariate analyses.

**Results:**

Seventy patients were included in the study. Median postoperative follow-up was 37 months (range 1–119). Median DFS and OS were 15.2 and 62.7 months, and 5-year DFS and OS rates were 16% and 53%. In univariate analysis, timing of metastasis presentation/treatment (combined colorectal and liver resection, “bowel first” approach or metachronous presentation) (*p* < 0.0001), ASA score (*p* = 0.003), chemotherapy after liver surgery (*p* = 0.028), T stage (*p* = 0.021), number of resected liver lesions (*p* < 0.0001), and liver margin status (*p* = 0.032) was significantly associated with DFS while peritoneal resection at colorectal surgery (*p* = 0.026), ASA score (*p* = 0.036), extension of liver resection (*p* = 0.024), chemotherapy after liver surgery (*p* = 0.047), and positive nodes (*p* = 0.018) with OS. In multivariate analysis, timing of metastasis presentation/treatment, ASA score, and chemotherapy (before and after liver surgery) resulted significantly associated with DFS and timing of metastasis presentation/treatment, positive nodes, peritoneal resection at colorectal surgery, and surgical approach (open or minimally invasive) of colorectal resection with OS.

**Conclusions:**

Surgery may provide good DFS and OS rates for CRC liver metastasis. Patient selection for surgery and correct timing of intervention within a multidisciplinary approach may be improved by taking into account negative prognostic factors which stress the importance of systemic therapy.

## 1. Introduction

Colorectal cancer (CRC) is the third leading cause of cancer-related death in developed countries [1]. The liver is the commonest site of distant spread of CRC, and liver metastases occur in up to 60% of those patients [2, 3].

Surgical treatment is the cornerstone of management of CRC, apart from staging. Nevertheless, some authors believe in a promoting effects of surgery on tumor spread stating that manipulation of the tumor and its vessels may promote tumor spillage, production of growth factors, and reduction of the release of antiangiogenic factors [4, 5]. On the contrary, other authors suggest that removal of at least great majority of tumor burden may reduce proinflammatory effects and the release of circulating malignant cell leading to a better control of metastasizing cells from host immunity [6, 7]. Due to the technical and technological improvements in liver surgery and perioperative care, hepatic resection has become at least a part of the standard of care in metastatic CRC. Indications to liver resection have been widened along the past 3 decades maintaining acceptable morbidity and mortality rates [8]. Nowadays, patients are considered suitable for surgery if all the disease can be resected with negative margins and preserving an adequate liver remnant [9, 10]. Unfortunately, only about 25% of the patients affected meets these criteria [3]. Moreover, a multimodality and multidisciplinary evaluation and treatment, when appropriate, is of paramount importance in this selected group of metastatic patients in order to provide the best chances of cure [11, 12].

In patients fit for surgery, resection may provide 5-year survival rates of 40–74% [9, 11, 12], 10-year survival rates of 16–23%, and a cure rate of 20% [10] compared to a 5-year survival rate of about 5% in case of palliative treatments [3]. On the other hand, recurrence rate is reported to be high (60–80%) with a 10–15% of early recurrence and disease-specific deaths [8].

In order to help in the selection of the more appropriate treatment, some prognostic factors in patients suffering from CRC and metastatic CRC have been identified [2, 3, 8, 10]. The aim of this study is to verify and analyze different factors which may affect disease-free survival (DFS) and overall survival (OS). They are either related to the patient, to the primitive CRC, or liver metastasis in the selected group of patients who underwent liver resection for first and isolated recurrence of colorectal cancer. Its perspective is the identification of the subgroups of patients who could benefit more from surgical resection in order to improve patient selection and the choice of adequate timing for liver resection within a multimodality treatment.

## 2. Materials and Methods

### 2.1. Study Design and Patients

From February 2006 to February 2018, all the patients affected by “liver only” first metastasization from CRC who underwent potentially curative surgical resection at the Hepatobiliary Surgery Unit of Careggi Teaching Hospital were included in the study. Patients undergoing intraoperative radiofrequency ablation (RFA) with a curative intent were also included. Patients with a primary rectal squamocellular carcinoma were excluded. Preoperative workup included triple phase-contrast enhanced computed tomography (CT) scan and pancolonoscopy. Liver volume assessment was performed when indicated. Magnetic resonance and positron emission tomography (PET) scan were used to rule out doubtful cases. Intraoperative ultrasound sonography (IOUS) was routinely used during liver surgery. Follow-up was done according to a standardized scheduled program including CT scan or abdominal ultrasound, colonoscopy, and blood test examination. It could be modified according to oncologist's indications. Retrieval of follow-up data was completed including the revision of any available medical records and phone call interviews.

Day of liver surgery was chosen as reference date. Disease-free interval was considered as the time between liver surgery and the diagnosis of any site of recurrence of disease or until the date of death while overall survival was considered as time between the liver surgery and the date of death or the last visit for alive patients. Recurrences were treated with surgery, chemotherapy, radiotherapy, percutaneous treatment, combinations of them, or best supportive care as appropriate.

According to timing of metastasis presentation/treatment, patients were divided into 3 groups: “synchronous combined surgery” that included patients who underwent combined surgery for primary tumor and liver metastasis, “synchronous bowel first” that included patients with metastatic disease from the beginning of their neoplastic history but liver metastases were not treated during colorectal surgery, and “metachronous” that included patients who developed liver metastasis after colorectal cancer surgery. The decision to perform combined or delayed surgery in synchronous presentation with or without any perioperative chemotherapy was discussed during Hospital Tumor Board meetings. Patient's conditions (i.e., comorbidities, bowel obstruction) and wishes, number, dimension, and position of the liver metastases at preoperative examination (confirmed or not at surgery time) were taken into account. Right colon comprehended lesions located from the cecum to transverse; left colon included also lesions located in the rectum. In univariate analysis, converted procedures were grouped with open surgery because this study is not an “intention-to-treat” analysis. Peritoneal resection was defined as any resection of the anterior aspect of the peritoneum macroscopically adherent/infiltrated by the primary tumor. Major hepatectomies were defined as resection of at least 3 segments according to Brisbane's classification [13]. Postoperative complications occurred after hepatic resections were evaluated if classified as at least grade 3 or 4 according to Clavien-Dindo classification [14]. Chemotherapy before and after liver surgery was considered if administered to the patient despite the interruption of the initially scheduled program. The lesion size (maximum diameter for both primary and metastatic tumors) and number of hepatic metastases resected were retrieved from histopathological response. T stage was classified according to American Joint Committee on Cancer (AJCC) TNM staging system, 7th edition definition [15]. Positive liver margins were defined in presence of neoplastic cells within the surface of resected liver.

### 2.2. Analysis

Patients' data were prospectively collected into a database which was retrospectively reviewed. Continuous variables were reported as median and range while categorical variables were reported as frequency and percentage. Differences between the three groups were analyzed using the Kruskal-Wallis test for continuous variables, while categorical variables were compared using the *χ*^2^ or Fisher exact test when appropriated. Statistical significance was defined as *p* value ≤ 0.05.

For univariate analysis, estimate of DFS and OS rates was calculated according to the Kaplan-Meier methods and compared using log-rank test. Hazard ratios (HR) and their 95% confidence intervals (CI) were calculated by means of the Cox proportional hazard model. The multivariate Cox regression model was used to evaluate the independent effect on DFS and OS of any factors whose *p* value was ≤0.15 at the univariate analysis. Parameters related to the histopathological response on liver specimens (i.e., number of resected lesions, maximum diameter, and liver margin status) were not considered in multivariate analysis because of their unavailability in the 7 patients who underwent intraoperative curative RFA alone in order to avoid their exclusion from this analysis.

Data were analyzed using the statistical software SAS version 9.2 (SAS Corporation, Cary, NC).

## 3. Results and Discussion

### 3.1. Results

#### 3.1.1. Patient Characteristics

Overall, 70 patients were included in the study. Median follow-up was 37 months (range 1–119). In particular, median follow-up among survivors was 48 months (range 2–116). Analyzed patient characteristics are displayed in Table 1. Patients undergoing combined surgery were 25, a two-step surgery was performed in 14 patients, and metachronous presentation of metastases was seen in 31 patients. Age, sex, and primary tumor distribution were similar within these three groups. Chemotherapy before liver surgery was administered in a very low percentage (8%) of “combined surgery” group (*p* < 0.0001).

Perioperative results are shown in Table 2. Open surgery technique (*p* = 0.006) and minor/parenchymal-sparing liver resection (*p* = 0.024) were more frequently applied in “combined surgery” group. ASA score and postoperative complication distribution in the 3 groups were similar with an overall median rate of severe complications after liver surgery of 14.3%. A statistically significant difference in the 3 groups was found for the administration of chemotherapy after liver surgery (*p* = 0.032) with the lowest percentage (28.6%) in “bowel first” group.

Analyzed histopathological results are shown in Table 3. T4 stage (*p* = 0.065) and bigger primary tumors (*p* = 0.024) were more frequently (*p* = 0.065) found in “bowel first” group. Lower median number (*p* = 0.334) and smaller size of liver metastases were reported in “combined surgery” group specimens (24 mm versus 35 mm in the other 2 groups, *p* = 0.005). Higher frequency of positive liver margins (*p* = 0.015) was found in “bowel first” group. Tumor grading was not evaluated since almost all the patients presented a G2 primary tumor. KRAS or BRAF evaluation was not available in 55.7% of the patients; consequently, this variable was not analyzed. However, 24 patients had a RAS wild-type while 15 patients presented a RAS mutation.

#### 3.1.2. Factors Associated with Disease-Free Survival (DFS) and Overall Survival (OS)

Recurrence after liver surgery was documented in 46 patients (66%). Early recurrence (within 6 months) occurred in 15 patients (21.4%), recurrence rate within the first year was 37% (26 patients), and no other recurrences were found after the third year from liver surgery. Median time between recurrence and death was 29 months (range 1–89 months). Ninety-day mortality was 1.4%.

Overall, 1-, 3-, and 5-year DFS rates were 59%, 17%, and 16%, respectively, with a median DFS rate of 15.2 months (95% CI 11.2–21.5). Overall, 1-, 3-, and 5-year OS rates were 94%, 68%, and 53%, respectively, with a median OS rate of 62.7 months (95% CI 43.7–67.8).

Results of univariate analysis of factors associated with DFS and OS are shown in Table 4.

**Figure 1:**
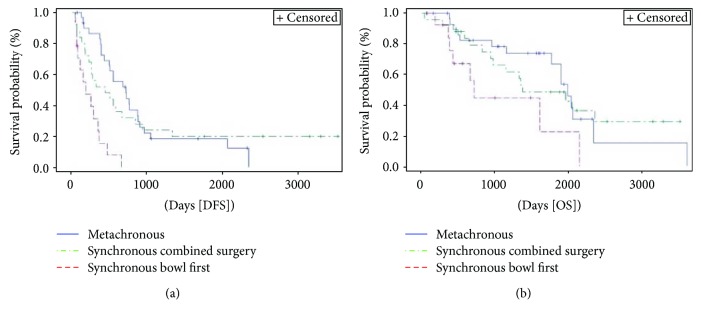
Kaplan-Meier curve of disease-free survival (DFS) (*p* < 0.0001) and overall survival (OS) (*p* = 0.085) stratified by timing of metastases presentation/treatment. Median DFS and OS for patients with synchronous presentation and combined surgery were 14.4 months (95% CI 7.2–27.8) and 45.3 months (95% CI 31–not evaluable [NE]), respectively, versus 6 (95% CI 2–11.4) and 23.6 months (95% CI 11.8–70.9), respectively, for those with synchronous presentation treated with a two-step surgery versus 23.5 (95% CI 14–28.8) and 65.7 months (95% CI 58.2–77.2), respectively, for patients with metachronous presentation.

Disease presentation and treatment timing (Figure 1) significantly affected DFS (*p* < 0.0001) but not OS (*p* = 0.085). Metachronous group had a median DFS of 23.5 months compared to 14.4 months of DFS for “combined surgery” group and 6 months for “bowel first” group. Compared to “combined surgery group”, “bowel first” group had an HR of 3.9 (95% HR CI 1.8–8.2) for DFS.

**Figure 2:**
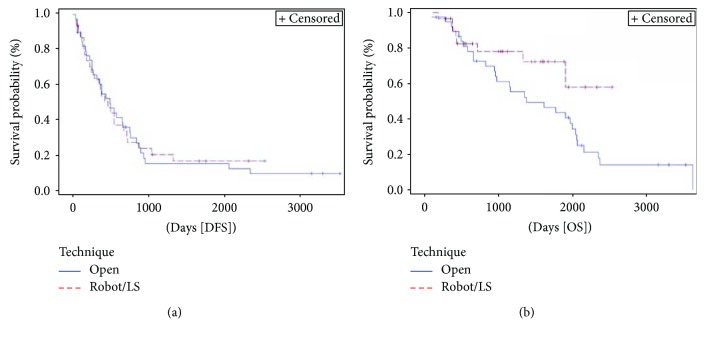
Kaplan-Meier curve of disease-free survival (DFS) (*p* = 0.885) and overall survival (OS) (*p* = 0.058) stratified by technique used to perform colorectal resection. Median DFS and OS for patients undergoing open and converted to open surgery techniques were 16.2 months (95% CI 8.9–24.8) and 45.3 months (95% CI 31.3–67.1), respectively, versus 15.2 (95% CI 8.3–23.5) and NE, respectively, for those undergoing minimally invasive surgery. Open = open surgery approach and “converted to open surgery”; robot/LS = minimally invasive approach including robotic and laparoscopic surgery; NE = not evaluable.

Surgical approach for colorectal surgery (Figure 2) resulted marginally significant for OS (*p* = 0.058). Compared to open and converted surgery, minimally invasive techniques had an HR of 0.5 (95% HR CI 0.2–1) for OS. Peritoneal resection at colorectal surgery was a negative significant factor for OS (*p* = 0.026) but not for DFS (*p* = 0.414).

**Figure 3:**
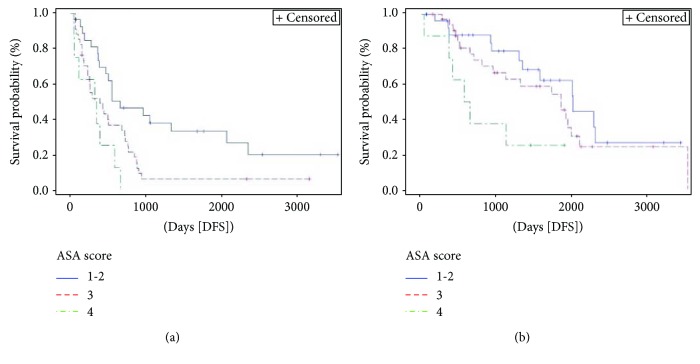
Kaplan-Meier curve of disease-free survival (DFS) (*p* = 0.003) and overall survival (OS) (*p* = 0.036) stratified by American Society of Anesthesiologists (ASA) score. Median DFS and OS for patients with ASA score 1-2 were 21.5 months (95% CI 12.4–67.8) and 67.8 months (95% CI 45.3–not evaluable [NE]), respectively, versus 12.6 (95% CI 7.2–22.4) and 62.6 months (95% CI 32–67.1), respectively, for those with ASA score 3 versus 10.9 (95% CI 1.3–19.2) and 20.5 months (95% CI 1.3–NE), respectively, for patients with ASA score 4.

ASA score (Figure 3) was significantly associated with both DFS (*p* = 0.003) and OS (*p* = 0.036). Three-year DFS and OS rates for ASA score 1-2 were 38% and 79%, respectively, compared with 6% and 67%, respectively, for ASA score 3 and 0% and 38%, respectively, for ASA score 4.

**Figure 4:**
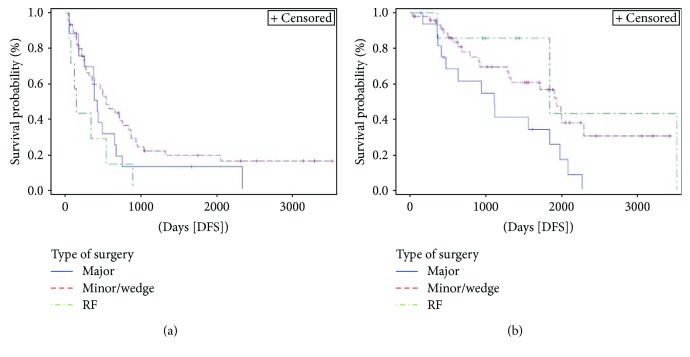
Kaplan-Meier curve of disease-free survival (DFS) (*p* = 0.060) and overall survival (OS) (*p* = 0.024) stratified by extension of liver resection. Median DFS and OS for patients undergoing major hepatectomies were 14 months (95% CI 6–21.5) and 37.9 months (95% CI 14–62.7), respectively, versus 18 (95% CI 10.4–27.8) and 65.7 months (95% CI 43.7–NE), respectively, for those undergoing a minor/wedge resections versus 5 (95% CI 1.5–18) and 62.6 months (95% CI 12.4–119.3), respectively, for patients treated with radiofrequency ablation. Major = major hepatectomies; minor/wedge = minor hepatic resection/wedge resection; RF = radiofrequency ablation; NE = not evaluable.

Extension of hepatic resection (Figure 4) resulted associated with OS (*p* = 0.024) and approached statistical significance in DFS (*p* = 0.060). Patients treated with RF had the worst DFS with 1-year DFS rate of 29% and an HR 2.5 (95% HR CI 1.1–5.7) for DFS if compared to patients treated with wedge/minor hepatic resection. Anyway, 1-year OS for patients treated with RF was 100%. Compared to minor/wedge resections, major hepatectomies had an HR 2.3 (95% HR CI 1.2–4.5) for OS.

**Figure 5:**
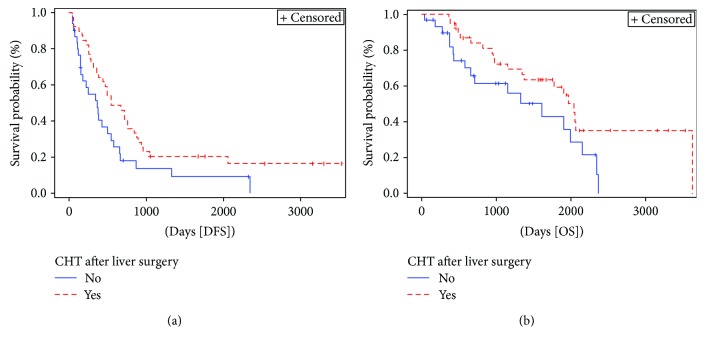
Kaplan-Meier curve of disease-free survival (DFS) (*p* = 0.028) and overall survival (OS) (*p* = 0.047) stratified by administration of chemotherapy after liver surgery. Median DFS and OS for patients not receiving chemotherapy were 12 months (95% CI 5–18.1) and 53 months (95% CI 19.2–70.9), respectively, versus 18 (95% CI 11.8–27.8) and 67.1 months (95% CI 44.4–119.3), respectively, for those receiving chemotherapy. CHT = chemotherapy.

Administration of chemotherapy after liver surgery (Figure 5) resulted positively associated with DFS (*p* = 0.028) and OS (0.047). Median time between liver surgery and start of chemotherapy treatment was 6.5 weeks (range 2.1–14.1).

**Figure 6:**
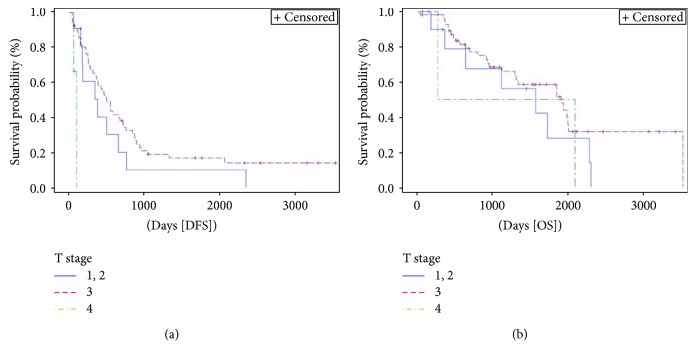
Kaplan-Meier curve of disease-free survival (DFS) (*p* = 0.002) and overall survival (OS) (*p* = 0.347) stratified by T stage^∗^. Median DFS and OS for T1-2 stages were 12.4 months (95% CI 5–21.6) and 53 months (95% CI 6–77.2), respectively, versus 16.6 (95% CI 11.8–23.5) and 64.7 months (95% CI 43.7–67.7), respectively, for T3 stage versus 3 (95% CI 2–3.3) and 40 months (95% CI 9.2–70.9), respectively, T4 stage. ^∗^T stage of primary tumor classified according AJCC TNM staging system.

**Figure 7:**
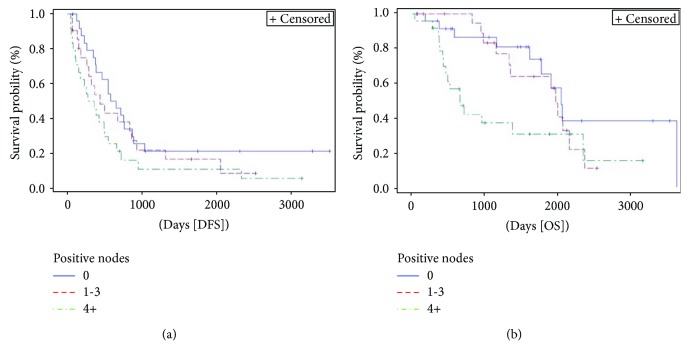
Kaplan-Meier curve of disease-free survival (DFS) (*p* = 0.098) and overall survival (OS) (*p* = 0.018) stratified by positive nodes. Median DFS and OS for patients without positive nodes were 20.3 months (95% CI 12.4–28.8) and 67 months (95% CI 53–119.3), respectively, versus 14.4 (95% CI 5.8–28.7) and 64.7 months (95% CI 37.9–70.9), respectively, for those with 1 to 3 positive lymph nodes versus 10.4 (95% CI 3.6–16.6) and 21.7 months (95% CI 14–77.2), respectively, for patients 4 or more positive lymph nodes.

T stage (Figure 6) was found to be a prognostic factor for DFS (*p* = 0.002). T3 stage had an HR 0.65 (95% HR CI 0.3–1.3) for DFS when compared to T1 and 2 stages. Number of positive nodes (Figure 7) was significantly associated with OS (*p* = 0.018). In case of no positive nodes, 3-year OS rate was 86% compared to 83% with 1–3 positive nodes and 37% with at least 4 positive nodes.

**Figure 8:**
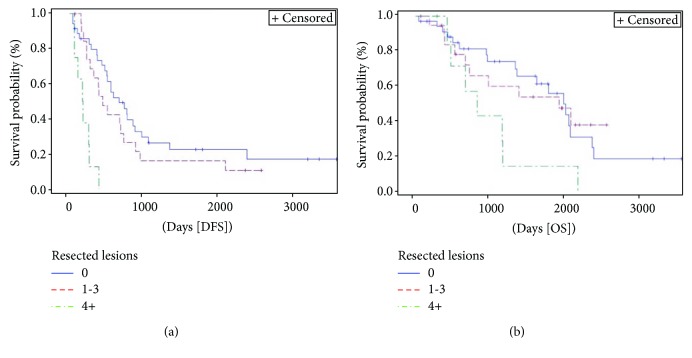
Kaplan-Meier curve of disease-free survival (DFS) (*p* = 0.0001) and overall survival (OS) (*p* = 0.066) stratified by number of resected metastatic lesions. Median DFS and OS for patients with 1 metastasis were 21.6 months (95% CI 15.2–28.7) and 64.7 months (95% CI 43.7–77.2), respectively, versus 14.4 (95% CI 7.3–23.5) and 62.7 months (95% CI 21.7–NE), respectively, for those with 2 or 3 metastases versus 5.7 (95% CI 1.9–8.5) and 27.1 months (95% CI 13.9–38.1), respectively, for patients with 4 or more metastases. NE = not evaluable.

**Figure 9:**
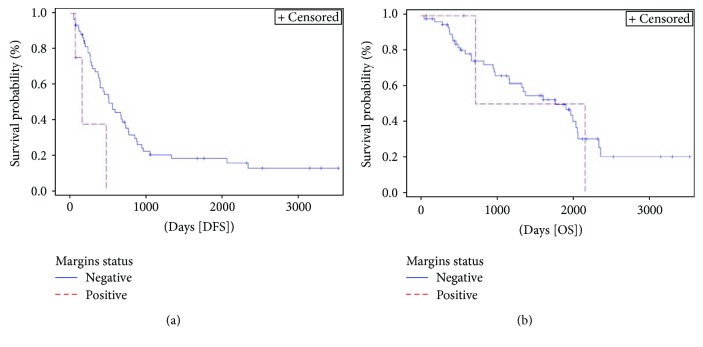
Kaplan-Meier curve of disease-free survival (DFS) (*p* = 0.032) and overall survival (OS) (*p* = 0.859) stratified by liver margin status. Median DFS and OS for patients with negative margins were 16.5 months (95% CI 12–23.5) and 58.2 months (95% CI 37.9–67.5), respectively, versus 4.8 (95% CI 2–15.2) and 47.2 months (95% CI 23.6–70.1), respectively, for those having positive margins.

Number of liver resected lesions (Figure 8) significantly affected DFS (*p* < 0.0001). For OS, a statistically significant difference was approached (*p* = 0.066). Patients with a single liver metastasis had a 3-year DFS rate of 26% compared to 16% and 0% for patients with 2-3 or 4 or more lesions, respectively. Patients with 4 or more liver lesions had a HR 7.4 (95% HR CI 3–18.4) for DFS when compared to patients with only one liver lesion. Positive liver margins (Figure 9) significatively affected DFS (*p* = 0.032). Patients with positive liver margin had an HR 3.5 (95% HR CI 1–11.6) for DFS if compared to the other patients.

Results of multivariate analysis for DFS and OS are displayed in Tables 5 and 6, respectively. Metachronous presentation, lower ASA score, and administration of chemotherapy (both before and after liver surgery) resulted significantly associated with a longer DFS. Synchronous presentation treated with combined surgery, absence of positive nodes, no peritoneal resection during colorectal surgery, and minimally invasive techniques used to perform colorectal resection were found to be significantly associated with longer OS.

### 3.2. Discussion

Several prognostic factors for DFS and OS in patients affected by liver metastasis from CRC have been found in this study. Although score systems considering different prognostic factors for CRC already exist [10], this study was conceived to investigate the role of potential prognostic factors related to the patients and either to primary tumor and liver metastasis in a restricted and recently treated cohort of patients. Study group included a consecutive series of patients suffering from liver metastasis as first recurrence of disease and undergoing potentially curative resection of liver metastases.

Time of metastatic presentation was a significant prognostic factor in both uni- and multivariate analyses. Patients with synchronous presentation treated with a two-step surgery had the worst prognosis. This group of patients was older, more frequently presenting with bowel obstruction and more comorbidities, with higher percentage of rectal localization of the primary tumor (57% versus 12%) and bigger lesions (median maximum size of 54 mm versus 35 mm). On the contrary, patients undergoing combined surgery received more frequently a parenchymal-sparing operation for smaller and for a median lower number of liver lesions. Obviously, surgery time was significantly longer in “combined surgery” group since surgery included also colorectal resection. However, no negative effects were determined on incidence of postoperative severe complications or on prognosis. Correct timing of resection, especially in synchronous presentation, and unequivocal criteria for surgery are still under debate [3]. In a recently published English population-based study, Vallance et al. [16] demonstrated an increase in number of combined surgery performed along the years, mostly since 2010. Patients fit for surgery, primary tumor not located in the rectum and superficial and unilobar metastases are the best conditions in which to perform a combined surgery without increase in morbidity and mortality rates [16, 17].

The site of primary tumor did not represent a significant prognostic factor in this series. A slight better prognosis was found for left-sided tumors. On the contrary, recent evidences show a worse prognosis for right colon cancer compared to left colon cancer and the relation between side and genetic alterations, molecular profile, and, consequently, response to chemotherapy [18, 19]. Prognostic relevance of the primary tumor side with a more indolent biology of left-sided cancer was also confirmed in the subgroup of metastatic patients [20, 21]. In the present study, rectal cancer that may have different prognosis was included within left colon group. However, there are previous published reports confirming a better prognosis for patients with liver metastasis from left colon and rectal cancer considered together [3, 22, 23]. Unfortunately, RAS and BRAF status evaluation was unavailable for a great part of this study group (44.3%) precluding further analysis of this parameter. However, RAS mutation was documented in 15 patients and 10 of them had a left-sided tumor confirming the relevance of molecular feature for the prognosis more than side of the tumor itself.

Technique chosen to perform colorectal surgery was an independent prognostic factor for OS (*p* = 0.007) on multivariate analysis. Advent of minimally invasive surgery was followed by many reports demonstrating at least not inferiority of these new techniques in oncological outcomes [24, 25]. Nevertheless, the well-known advantages in postoperative outcomes of minimally invasive technique with reduction of surgical stress and better preservation of immune system [4], together with lower risk of incisional hernias and adhesions, may explain this benefit in OS rate.

Peritoneal resection at colorectal resection resulted strongly associated with OS rate in both univariate and multivariate analyses even though in the presence of wide 95% confidence intervals. This is consistent with previous findings that peritoneal wound seems to be predictive of the alterations in the immune response more than skin incision [4]. Anyway, the small sample of patients who received a peritoneal resection should lead to a careful and critical analysis of these results.

ASA score resulted a prognostic factor for DFS in both uni- and multivariate analyses and for OS in univariate analysis. It seems quite obvious that patients in worst conditions may have a worse response against the tumor and have higher chance to die for any cause. Careful selection of ASA 4 patients suitable for surgery is recommended.

In univariate analysis, extension of liver resection approached statistical significance for DFS (*p* = 0.060) and resulted significantly associated with OS (*p* = 0.024). Radiofrequency ablation did not provide equivalent DFS rates when compared to resections. However, beyond intrinsic technical limits, this treatment was used in selected and fragile patients with smaller lesions that may be associated with multiple micrometastasis explaining lower DFS rates consistent with previous literature reports [26]. On the other hand, lesions may be completely treated, and in case of recurrence, procedure may be repeated with a minimum impact on the patients and on liver function explaining encouraging OS rates. Patients undergoing minor hepatic resection or wedge resection had a better prognosis compared to major hepatectomies with a 5-year OS rate of 57% compared to 34%. Parenchymal-sparing operations are becoming the standard of care for CRC liver metastasis even in light of possible liver relapse of disease that may require redo surgery with curative possibilities [27, 28]. Several papers reported the advantages of this technique in terms of reduced postoperative morbidity and some survival benefit with adequate oncological outcomes. In consideration of these results, parenchymal-sparing surgery should be preferred, whenever technically feasible [29, 30].

Chemotherapy before liver surgery resulted a negative prognostic factor for DFS in multivariate analysis in the present study. Vigano et al. [8] proposed to evaluate patient response between end of chemotherapy and liver resection as a “time test” and a prognostic factor suggesting the possibility to exclude from surgery about a 15% of patients who would present early recurrence. However, their findings deserve further evaluations. On the contrary, in this series, administration of chemotherapy after liver surgery resulted a positive prognostic factor for DFS and OS in univariate analysis and for DFS in multivariate analysis. Median time between liver surgery and initiation of chemotherapy was 6.5 weeks (range 2.1–14.1). Obviously, there is a group of patients who could be indicated for adjuvant treatments because of the stage of disease, but they are not considered fit for them. This is mostly related to older age and comorbidities such as previous heart disease. All these factors may be related to the worst OS rate more than chemotherapy itself, coherently with multivariate analysis results. Previously published papers reported improved outcomes after adjuvant chemotherapy without an increase in OS [10, 31] confirming also that elderly patients are often oncologically undertreated [32]. Nevertheless, initiation of chemotherapy within 6–8 weeks is a recognized prognostic factor for OS [33, 34]. Unfortunately, the small sample of patients receiving chemotherapy after surgery in this series precluded a subgroup analysis of the impact of the different chemotherapy regimens and use of molecular targeted therapies.

T stage but not tumor size resulted a prognostic factor for DFS on univariate analysis. While T4 stage resulted associated with very low DFS and OS rates after liver surgery, interestingly, T3 stage had a better prognosis if compared to T1-2 stages. This may be related to the different administrations of chemotherapy being T1-2 stages usually not indicated for chemotherapy. This explanation is consistent with the results of multivariate analysis in which chemotherapy but not T stage resulted independent prognostic factor for DFS. Positive nodes resulted significantly associated with OS in uni- and multivariate analyses. These findings underline the prognostic impact of AJCC TNM classification. Careful selection for liver surgery in patients with a primary tumor T4 stage or N2 is recommended.

Number of resected liver lesions was significantly associated with DFS. On the contrary, size of resected liver lesions did not affect prognosis in this series. Interestingly, intermediate size of liver lesions showed better DFS rates when compared with smaller or bigger lesions. A possible explanation may be that in case of small lesions, presence of multifocal undetectable micrometastases is possible while bigger lesions are related to a huge burden of disease.

Liver margin status was an independent prognostic factor for OS, accordingly to the prognostic importance of at least submillimetric clear margin which has been previously reported [35].

This study has some limitations. It is a retrospective study with the inherent selection bias. It is a small series leading to a careful and critical interpretation of some findings. Nevertheless, because of the small sample available, some variables analyzed were divided into subgroup that may include patients with different prognosis related to that variable (i.e., left colon including rectum or administration of chemotherapy without distinction if a molecular targeted therapy was added or not). A strength of this paper is that a recent series of resected patients has been analyzed, but on the other hand, follow-up period is quite short considering the proposal of at least 10-year follow-up due to the possibility of late recurrence [10]. Furthermore, although follow-up scheduled program was standardized, some patients may be evaluated with a different timing and patient compliance was not always complete. Consequently, date of recurrence may be influenced.

## 4. Conclusions

In the treatment of liver metastases from CRC, several factors were associated with at least marginal significance with either DFS or OS. Synchronous presentation treated with combined surgery and metachronous presentation, use of a minimally invasive technique in colorectal surgery, no necessity to perform a peritoneal resection in colorectal surgery, minor/wedge liver resection, administration of chemotherapy after liver surgery, T1–3 stages, negative lymph nodes, single liver lesions and negative liver margins were related with better prognosis. Moreover, none of the analyzed factor was associated with a so bad prognosis to contraindicate surgery.

Multimodality and multidisciplinary treatment is of paramount importance to achieve higher cure rates, and in this light, DFS should be the most important parameter to evaluate. Aggressive perioperative systemic treatment may be required in the presence of negative prognostic factors, whenever possible. Nevertheless, patient selection remains challenging and further improvements in prognostication are necessary to identify patients unlikely to benefit from resection.

## Figures and Tables

**Table 1:** Patients' characteristics.

	Synchronous combined surgery (*n* = 25) (35.7%)	Synchronous “bowel first” (*n* = 14) (20%)	Metachronous (*n* = 31) (44.3%)	Total (*n* = 70)	*p* value
Age (years, range)	68 (34–85)	75 (46–82)	70 (52–85)	69.5 (34–85)	0.730
Sex (*n*, %)					0.683
Male	15 (60%)	9 (64.3%)	16 (51.6%)	40 (57.1%)	
Female	10 (40%)	5 (35.7%)	15 (48.4%)	30 (42.9%)	
Bowel obstruction (*n*, %)	5 (20%)	7 (50%)	7 (22.6%)	19 (27.1%)	0.097
Site of primary tumor (*n*, %)					0.343
Right colon	8 (32%)	2 (14.3%)	11 (35.5%)	21 (30%)	
Left colon	17 (68%)	12 (85.7%)	20 (64.5%)	49 (70%)	
CHT before liver surgery	2 (8%)	11 (78.6%)	20 (64.5%)	33 (47%)	**<0.0001**

CHT = chemotherapy.

**Table 2:** Perioperative results.

	Synchronous combined surgery (*n* = 25) (35.7%)	Synchronous “bowel first” (*n* = 14) (20%)	Metachronous (*n* = 31) (44.3%)	Total (*n* = 70)	*p* value
*Colorectal surgery*					
Technique (*n*, %)					**0.006**
Open	15 (60%)	5 (35.7%)	7 (22.6%)	40 (57.1%)	
Minimally invasive	5 (20%)	9 (64.3%)	17 (54.8%)	31 (44.3%)	
Converted	5 (20%)	0	7 (22.6%)	12 (17.1%)	
Peritoneal resection (*n*, %)	2 (8%)	1 (7.1%)	2 (6.4%)	5 (7.1%)	1.00
*Liver surgery*					
ASA (*n*, %)					0.522
1	3 (12%)	1 (7.1%)	2 (6.4%)	6 (8.6%)	
2	7 (28%)	4 (28.6%)	11 (35.5%)	22 (31.4%)	
3	11 (44%)	6 (42.9%)	17 (54.8%)	34 (48.6%)	
4	4 (16%)	3 (21.4%)	1 (3.2)	8 (11.4)	
Type of surgery (*n*, %)					**0.024**
Minor/wedge	22 (88%)	7 (50%)	17 (54.8%)	46 (65.7%)	
Major	3 (12%)	4 (28.6%)	10 (32.3%)	17 (24.3%)	
RFA	0	3 (21.4%)	4 (12.9%)	7 (10%)	
Surgery duration (min, range)	300 (170–145)	242.5 (175–369)	230 (50–315)	255 (50–450)	<0.0001
Complications (CD III-IV) (*n*, %)	3 (12%)	3 (21.4%)	4 (12.9%)	10 (14.3%)	0.743
CHT after liver surgery (*n*, %)	18 (72%)	4 (28.6%)	17 (54.8%)	39 (55.7%)	**0.032**

ASA = American Society of Anesthesiologists; Minor/wedge = minor hepatectomies/hepatic wedge resections; Major = major hepatectomies; RFA = radiofrequency ablation; CD III-IV = Clavien-Dindo classification grade III-IV; CHT = chemotherapy.

**Table 3:** Histopathological results.

	Synchronous combined surgery (*n* = 25) (35.7%)	Synchronous “bowel first” (*n* = 14) (20%)	Metachronous (*n* = 31) (44.3%)	Total (*n* = 70)	*p* value
*Colorectal specimen*					
Size (mm, range)	35 (17–130)	54 (25–90)	35 (4–82)	40 (4–130)	**0.024**
T stage^∗^ (*n*, %)					0.065
1-2	2 (8%)	4 (28.6%)	5 (16.1%)	11 (15.7%)	
3	22 (88%)	8 (57.1%)	26 (83.9%)	56 (80%)	
4	1 (4%)	2 (14.3%)	0	3 (4.3%)	
Nodes harvested (*n*, range)	17 (7–76)	25 (6–48)	26 (9–50)	22 (6–76)	
Positive nodes (*n*, range)	2 (0–10)	3.5 (0–17)	1 (0–12)	2 (0–17)	0.217
Mucinous histotype	3 (12%)	1 (7.1%)	9 (29%)	13 (18.6%)	0.159
*Liver specimen*	*n* = 25	*n* = 11	*n* = 27	*n* = 63	
Resected lesions (*n*, range)	1 (1–11)	2 (1–6)	1 (1–4)	1 (1–11)	0.334
Size (mm, range)	24 (4–50)	35 (15–80)	35 (12–110)	30 (4–110)	**0.005**
Positive margins (*n*, %)	0	3 (27.3%)	1 (3.7%)	4 (6.35)	**0.015**

^∗^T stage according to TNM definition AJCC 7th edition.

**Table 4:** Univariate analysis of factors associated with disease-free and overall survival.

	*n*	DFS					OS				
		1 y (%)	3 y (%)	5 y (%)	Median (months, CI)	*p* value	1 y (%)	3 y (%)	5 y (%)	Median (months, CI)	*p* value
Timing of metastases presentation/treatment						**<0.0001**					0.085
Synchronous combined	**25**	52	24	20	14.4 (7.2–27.8)		92	66	48	45.3 (31–NE)	
Synchronous “bowel first”	**14**	16	0	0	6 (2–11.4)		84	45	22	23.6 (11.8–70.9)	
Metachronous	**31**	83	19	19	23.5 (14–28.8)		100	78	67	65.7 (58.2–77.2)	
Age						0.648					0.552
<65	**21**	57	14	14	15.2 (7.2–22.1)		100	69	69	67.5 (23.6–77.9)	
≥65	**49**	59	19	16	16.2 (10.4–23.5)		91	68	46	53 (37.9–65.7)	
Sex						0.574					0.929
Male	**40**	59	14	11	16.4 (8.9–21.7)		95	75	57	64.2 (44.4–NE)	
Female	**30**	58	22	22	14 (8.3–23.7)		93	58	49	38.1 (27.1–119.3)	
Bowel obstruction						0.860					0.985
No	**51**	59	17	15	16.4 (10.4–21.7)		92	69	52	62.6 (43.7–70.9)	
Yes	**19**	56	17	0	12.7 (4.2–28.7)		100	64	57	65.7 (21.7–119.3)	
Site of primary tumor						0.488					0.661
Right colon	**21**	60	20	20	16.2 (8.5–29.4)		90	53	41	37.9 (17.1–77.2)	
Left colon	**49**	58	16	13	15.2 (9.4–19.2)		96	74	60	64.7 (44.4–67.8)	
CHT before liver surgery						0.080					0.531
No	**37**	62	23	20	19.2 (8.5–28.7)		92	71	52	62.6 (37.9–77.2)	
Yes	**33**	54	10	10	12.7 (9.4–18)		97	63	58	62.7 (31.1–70.9)	
*Colorectal surgery*											
Technique						0.885					0.058
Open and converted	**39**	60	15	15	16.2 (8.9–24.8)		92	61	44	45.3 (31.3–67.1)	
Minimally invasive	**31**	57	20	16	15.2 (8.3–23.5)		97	78	72	NE (43.7–NE)	
Peritoneal resection						0.414					**0.026**
No	**65**	60	17	15	16.4 (11.8–21.7)		97	72	56	62.7 (44.3–67.8)	
Yes	**5**	40	20	20	3.3 (1.3–NE)		60	20	20	12.4 (1.3–NE)	
*Liver surgery*											
ASA						**0.003**					**0.036**
1-2	**28**	73	38	33	21.5 (12.4–67.8)		92	79	62	67.8 (45.3–NE)	
3	**34**	52	6	6	12.6 (7.2–22.4)		97	67	54	62.6 (32–67.1)	
4	**8**	38	0	0	10.9 (1.3–19.2)		88	38	25	20.5 (1.3–NE)	
Type of surgery						0.060					**0.024**
Minor/wedge	**46**	59	22	19	18 (10.4–27.8)		93	69	57	65.7 (43.7–NE)	
Major	**17**	69	13	13	14 (6–21.5)		94	54	34	37.9 (14–62.7)	
RFA	**7**	29	0	0	5 (1.5–18)		100	86	86	62.6 (12.4–119.3)	
Surgery duration						0.999					0.945
<255 min	**34**	67	13	13	18 (12–25)		94	71	55	64.7 (38.1–67.8)	
≥255 min	**36**	51	21	18	12.4 (8.5–21.7)		94	65	51	62.6 (31.1–70.9)	
Complications (CD III-IV)						0.441					0.721
No	**60**	59	18	16	16.2 (10.4–22.1)		95	68	53	62.6 (38.1–67.5)	
Yes	**10**	58	12	12	12.6 (1.3–30.9)		89	67	53	77.2 (9.2–77.2)	
CHT after liver surgery						**0.028**					**0.047**
No	**31**	48	14	9	12 (5–18.1)		90	61	43	53 (19.2–70.9)	
Yes	**39**	67	20	20	18 (11.8–27.8)		97	72	50	67.1 (44.4–70.9)	
*Colorectal specimen*											
Size						0.510					0.188
≤33 mm	**26**	60	8	8	18 (8.9–21.7)		100	79	57	62.7 (45.3–70.9)	
34–49 mm	**20**	69	24	16	23.5 (7.3–29.4)		95	72	64	65.7 (32–119.3)	
≥50 mm	**24**	48	22	22	11.8 (6–15.2)		87	51	40	37.9 (12.6–67.8)	
T stage						**0.002**					0.347
1-2	**11**	51	10	10	12.4 (5–21.6)		90	68	28	53 (6–77.2)	
3	**56**	62	19	17	16.6 (11.8–23.5)		96	68	59	64.7 (43.7–67.7)	
4	**3**	0	0	0	3 (2–3.3)		50	50	50	40 (9.2–70.9)	
Positive nodes						0.098					**0.018**
0	**25**	75	21	21	20.3 (12.4–28.8)		96	86	65	67 (53–119.3)	
1–3	**21**	54	21	16	14.4 (5.8–28.7)		100	83	64	64.7 (37.9–70.9)	
4+	**24**	46	10	10	10.4 (3.6–16.6)		87	37	31	21.7 (14–77.2)	
Mucinous histotype						0.940					0.286
No	**57**	58	17	15	58.2 (38–67)		96	68	50		
Yes	**13**	60	17	17	(9–119.3)		84	67	67		
*Liver specimen*											
Resected lesions						**0.0001**					0.066
1	**35**	73	26	22	21.6 (15.2–28.7)		91	74	56	64.7 (43.7–77.2)	
2-3	**20**	63	16	16	14.4 (7.3–23.5)		95	60	54	62.7 (21.7–NE)	
4+	**8**	13	0	0	5.7 (1.9–8.5)		100	43	14	27.1 (13.9–38.1)	
Size						0.199					0.088
I tertile	**20**	55	20	15	17.2 (8.5–25)		90	73	50	60.4 (31.3–67.5)	
II tertile	**22**	73	25	25	22.5 (12–28.8)		95	75	60	NE (32–NE)	
III tertile	**21**	57	11	11	12.6 (5–24.8)		94	45	39	31.5 (14–70.9)	
Margin status						**0.032**					0.859
Negative	**59**	63	20	18	16.5 (12–23.5)		100	66	50	58.2 (37.9–67.5)	
Positive	**4**	38	0	0	4.8 (2–15.2)		93	50	50	47.2 (23.6–70.1)	

NE = not evaluable; CHT = chemotherapy; ASA = American Society of Anesthesiologists; Minor/wedge = minor hepatectomies/hepatic wedge resections; Major = major hepatectomies; RFA = radiofreqency ablation; CD III-IV = Clavien-Dindo classification grade III-IV. T stage according to TNM definition AJCC 7th edition.

**Table 5:** Multivariate analysis of factors associated with disease-free survival.

	*p* value	HR	HR and 95% CI
Timing of metastases presentation/treatment	**0.0008**		
Synchronous “combined surgery”	ref	ref	ref
Synchronous “bowel first”	*0.219*	1.9	0.7–5.5
Metachronous	*0.067*	0.5	0.2–1.1
ASA	**0.005**		
1-2	ref	ref	ref
3	*0.001*	2.7	1.5–4.9
4	*0.134*	2.1	0.8–5.5
CHT before liver surgery	**0.027**		
No	ref	ref	ref
Yes	*0.027*	2.5	1.1–5.6
CHT after liver surgery	**0.028**		
No	ref	ref	ref
Yes	*0.028*	0.5	0.2–0.9

ref = reference; ASA = American Society of Anesthesiologists; CHT = chemotherapy.

**Table 6:** Multivariate analysis of factors associated with overall survival.

	*p* value	HR	HR and 95% CI
Timing of metastases presentation/treatment	**0.053**		
Synchronous “combined surgery”	ref	ref	ref
Synchronous “bowel first” surgery”	*0.025*	2.8	1.1–7
Metachronous	*0.895*	1.1	0.5–2.3
Positive nodes	**0.008**		
0	ref	ref	ref
1–3	*0.267*	1.7	0.7–4.4
4+	*0.003*	3.8	1.6–9.1
Peritoneal resection	**0.0003**		
No	ref	ref	ref
Yes	*0.0003*	12.1	3.1–46.7
Technique for colon resection	**0.007**		
Open	ref	ref	ref
Minimally invasive	*0.007*	0.3	0.1–0.7

ref = reference.

## Data Availability

The data used to support the findings of this study are available from the corresponding author upon request.
